# Alleviation of nonalcoholic steatohepatitis induced by tetracycline in rats by *Coffee Arabica* extract through autophagy signals (mTOR/LC3-B)

**DOI:** 10.1038/s41598-026-43605-6

**Published:** 2026-03-27

**Authors:** Merehan Alaa-ElDin Mohamed, Said S. Moselhy, Shaimaa Rihan, Mustafa M. M. Elbakry

**Affiliations:** 1 Biochemistry Department, Faculty of Science, Ain Shams University, Cairo, Egypt; 2 Faculty of Biotechnology, October University for Modern Sciences and Arts (MSA), Cairo, Egypt

**Keywords:** Chlorogenic acid, *Coffee Arabica*, Docking, LC3-B, mTOR, Rats, Tetracycline, Biochemistry, Diseases, Drug discovery

## Abstract

The autophagy mechanism is a key point for liver protection against nonalcoholic steatohepatitis (NASH). By specifically selecting *Coffea arabica*, this study leverages its high concentration of chlorogenic acid to modulate autophagy, a critical cellular recycling process that is typically suppressed during the development of NASH-related liver damage. We investigated the impact of *Coffea Arabica* methanolic extract (CAME) on autophagy-related markers (mTOR and LC3-B) mediated abrogation of tetracycline (TET) induced NASH in rats. Sixty male albino rats weighing 150 ± 10 g were equally divided into six groups: group 1 (control) received a chow diet; group 2 (NASH) received TET orally (1 g/kg bw) for 8 days; group 3 (CAME) received *Coffea Arabica* methanolic extract (CAME) orally (100 mg/kg bw) for 28 days; group 4 (treatment) received TET then CAME treatment for 28 days; group 5 (preventive) received CAME (100 mg/kg) for 28 days then TET orally (1 g/kg) for 8 days; and group 6 (protective) received both TET and CAME orally for 8 days. ELISA technique was used to measure mTOR and LC3-B content in liver tissue homogenate. Moreover, transmission electron microscope analysis carried out to detect pathological alterations in liver tissue. Also, molecular docking analysis was done. *Coffea Arabica* methanolic extract analysis by GC/MS revealed that CAME contained the highest percentage of chlorogenic acid (12.7963%). The biochemical data obtained pointed out that the mTOR level was significantly increased (~71.62%) while LC3-B decreased (~28.08%) in the NASH group compared with control. Administration of CAME abrogated these abnormalities. Liver examination by electron microscope indicated improvement abnormalities caused by TET in treatment with CAME. Docking study showed that chlorogenic acid has binding energy  − 7.554 favorable to mTOR than ATP-γS. We concluded that CAME stimulated a protective mechanism against NASH via LC3B and mTOR modulation which should attract further research to confirm our results and fully understand its mechanism of induction.

## Introduction

Nonalcoholic steatohepatitis (NASH) is an advanced stage of fatty liver associated with inflammation, cellular damage, fibrosis and cirrhosis^1^. Tetracycline (TET) is a broad-spectrum anti-bacterial agent used in the treatment of different infections, but at higher doses, it causes toxicological effects such as nephrotoxicity, hepatotoxicity, and testicular damage in an animal model^2^. Experimentally, TET can be used for induction of NASH in animals^3^. Autophagy is a physiological mechanism that contributes significantly to the survival and maintenance of cells by breaking down cytoplasmic dead organelles, proteins, and macromolecules^4^.

From 25.26% in 1990–2006 up to 38% in 2016–2019, the prevalence of NAFLD rose by + 50.4% worldwide. According to current statistics, prevalence rates in Egypt are quite high, surpassing 40% in the overall population including 30% in young adults^5,6^.

An essential mechanism for lysosomes to break down intracellular components is autophagy. In particular, decreased autophagic activity may accelerate the onset of liver steatosis and its progression to liver damage. Through the Atg1Atg13 complex, mTOR activation prevents autophagy in its early stages. This pathway is linked to the development of NAFLD because of the intricate relationship between the autophagy process and the mTOR pathway^7,8^.

The autophagosome membrane is the main component of LC3-B’s function in autophagy. LC3-B on the inner surface of the phagophore engages with autophagy receptors to help construct the autophagosome membrane^9^. Autophagy promotes autophagosomes formation and engulfs cytoplasmic components such as cytosolic proteins and organelles^10^. On the surface of developing autophagosomes, cytosolic LC3-I conjugates to phosphatidylethanolamine generating LC3-II, a typical marker for autophagosomes^11^. Serine/threonine kinase, a mammalian target of rapamycin (mTOR), is a master regulator of metabolism within cells. Additionally, mTOR is essential for controlling autophagy^12,13^.

Coffee is the world’s most popular caffeine-containing beverage. It contains a complex mixture of phytocompounds including alkaloids, flavonoids and phenolics as chlorogenic acid, that have numerous health advantages as a powerful antioxidant^14^. In our previous study, we reported that, NASH induced rats treated with *Coffea Arabica* methanolic extract (CAME) decreased the activity of ALT, AST, and ALP and the levels of PT, PTT, total cholesterol, triglycerides, LDL-C, and VLDL-C accompanied by increasing the levels of albumin, total protein, HDL-C, and total antioxidant capacity in comparison with the NASH group^15^.

For NASH, there is no efficient therapy. Therefore, understanding pathogenic mechanisms causing disease start and progression is essential for developing innovative therapies. Since they offer new hope for NASH, natural constituents from medicinal plants are presently the focus of more research. An alternate and supplemental natural functional dietary supplement, such as Arabica coffee, which is high in chlorogenic acid and targets the autophagy process that mediates the amelioration effect of NASH, may be a viable agent^15^. Therefore, the current study aimed to investigate the modulation impact of *CAME* on autophagy-related markers (mTOR and LC3B) as a protective mechanism against NASH induced by TET in rats via the estimation of mTOR and LC3-B content in liver tissue homogenate using ELISA technique besides the transmission electron microscope analysis of liver, along with docking study of chlorogenic acid and mTOR as a supportive computational analysis.

## Materials and methods

### Identification of active components of *Arabica coffee* extract

Fresh *Coffea Arabica* beans were procured from the local traditional market in Cairo, Egypt (Batch # LT18M). It was grounded for 3 min using an electric mixer^16^, the ground samples were then extracted by soaking it in different solvents (80% methanol, 80% ethanol, and water) for 24 h at room temperature. The water extract was carried out according to Acidri et al.^17^, then the samples were filtered through Whatman No. 1 filter paper. The collected filtrates were dried under vacuum using a rotary evaporator.

### Silylation agent: N, O-Bis(trimethylsilyl)trifluoroacetamide (BSTFA) with trimethyl chlorosilane

The reaction was carried out by adding 300 uL of BSTFA + 300 uL of the sample after extraction and heating in a water bath at 80 °C for 2 h and after that it was injected into the GC/MS under the above conditions. The constituents were determined by mass fragmentations with The NIST mass spectral search program for the NIST/EPA/NIH mass spectral library Version 2.2.

The extract was subjected for chromatographic analysis using GC/MS (Agilent Technologies 7890B GC Systems combined with 5977 A Mass Selective Detector). The capillary column was used (HP-5MS Capillary; 30.0 m × 0.25 mm ID × 0.25 μm film) and the carrier gas used was helium at a pressure of 8.2 psi with 1 µL injection. The sample was analyzed with the column held initially for 6 min at 60 °C after injection, then the temperature increased to 300 °C with a 20 °C/ minutes heating ramp, with a 5 min hold. Injection was carried out in split mode (1:1) at 300 °C. MS scan range was (*m*/*z*): 50–550 atomic mass units (AMU) under electron impact (EI) ionization (70 eV) and solvent delay 8 min.

The extract ingredients percentage composition was represented as peak area percentage.

### Experimental animals design

Sixty male albino Wistar rats, weighing 150 g ± 10 g, were obtained from the animal farm of the Egyptian Holding Company for Biological Products and Vaccines (VACSERA), Helwan, Cairo, Egypt. Rats were maintained in a controlled environment conditions (12 h light/dark cycles and 21–25 °C) with free access to a normal chow diet as well as tap water.

The experiment was carried out in the animal house at the Department of Zoology, Faculty of Science, Ain Shams University according to ethical committee “The Research Ethics Committee, Faculty of Science, Ain Shams University approved the research protocol (ASU-SCI/BIOC/2023/4/1). All methods are reported in accordance with ARRIVE guidelines.

**Fig. 1:**
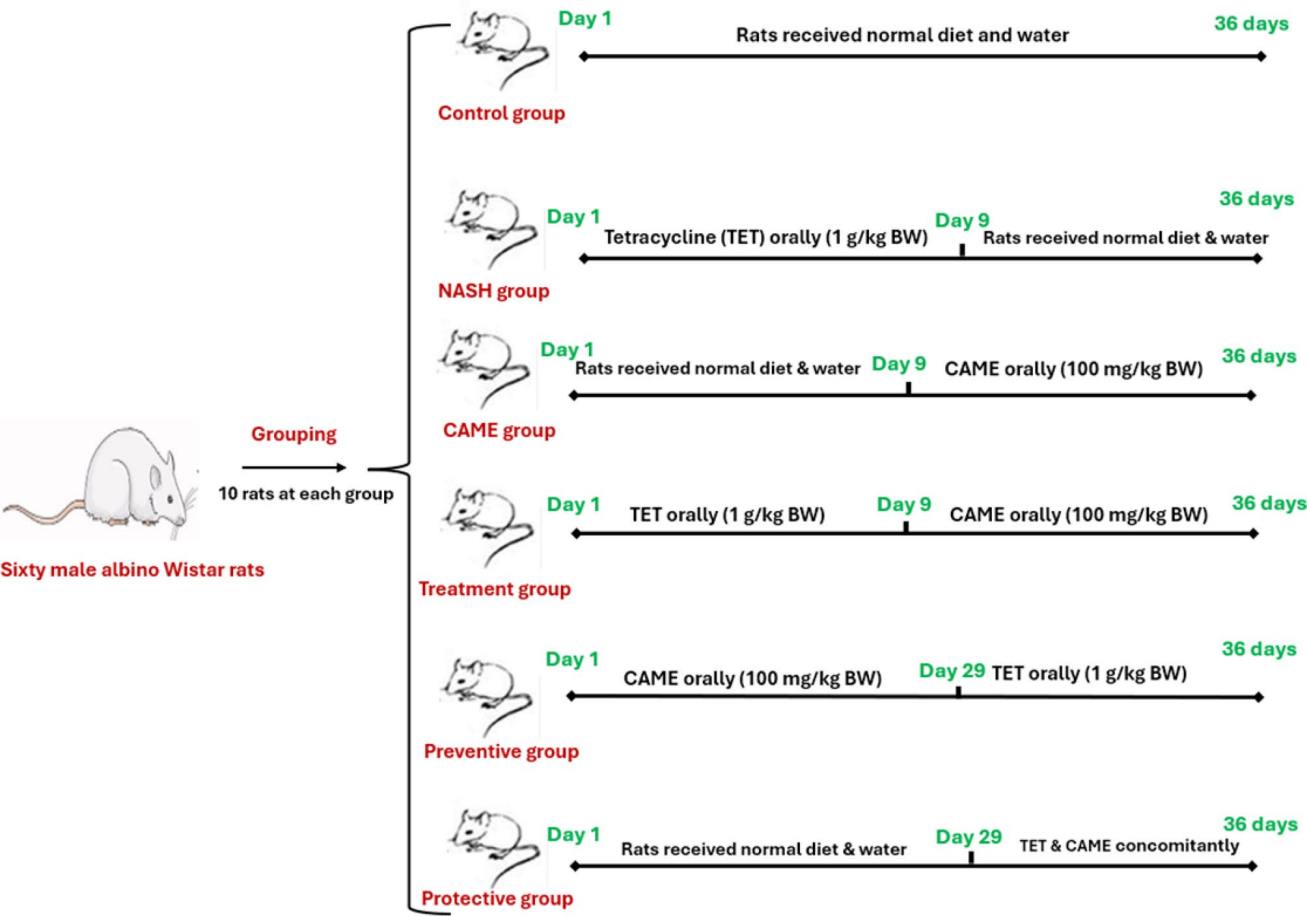
Schematic diagram of experimental design.

After 10 days for adaptation. Rats were randomly divided into six equal groups (*n* = 10) as shown in Fig. 1.

Group 1 (Control): Rats received a chow diet and tap water for 36 days.

Group 2 (NASH): Rats were given tetracycline (dissolved in water daily) at a dose of 1 g/kg bw orally for 8 days, according to Benet et al.^18^.

Group 3 (CAME): Rats were given *Coffee Arabica* methanolic extract at a dose (100 mg/kg b.w) orally starting from day 9, which is equivalent to two cups consumed by a man per day for 28 days, according to Al-Megrin et al.^19^.

Group 4 (Treatment): Rats were given TET orally (1 g/kg bw) for 8 days and then treated with CAME (100 mg/kg) orally for 28 days starting from day 9.

Group 5 (Preventive): Rats were given CAME (100 mg/kg bw) orally for 28 days as group 3, then received TET (1 g/kg bw) orally for 8 days.

Group 6 (Protective): Rats were concomitantly given TET (1 g/kg bw) and CAME (100 mg/kg bw) orally for 8 days starting from day 29.

### Samples collection

All methods were carried out in accordance with relevant guidelines and regulations. At the end of the experimental period (36 days), the animals were fasted for 24 h. The rats were euthanized by thiopental and dissected for tissue collection.

### Preparation of liver homogenate for the analysis of autophagy markers

The liver tissue was excised, cleaned in isotonic sterile saline, blotted dry with filter paper, and divided into two parts. One gram of liver was homogenized in (10% w/v) in phosphate-buffered saline pH 7.4 using a glass tissue homogenizer with a Teflon pestle. The whole liver homogenate was centrifuged at 18,000× g for 15 min at 4 °C using (Cooler Microfuge Laborzentrifugen, Sigma, Germany) to obtain the cytosolic fraction, which was then collected, aliquoted, and stored at -20 °C until further analyses for the measurement of mTOR level using a rat mTOR kit purchased from Cloud-Clone Corp. (USA) (cat. no. E-31091Ra) and LC3-B level using a rat LC3-B kit purchased from Cloud-Clone Corp. (USA) (cat. no. E-31077Ra).

### Pathological alterations examination by transmission electron microscope

The second part of the liver was cut into small pieces (1mm^2^ and fixed in ice-cold 3% glutaraldehyde, postfixed in 1% osmium tetroxide (1 h), then ethanol series dehydration occurred. After that, samples were embedded in epoxy resin (37 °C; 3 h), followed by ultra-thin sectioning (60–90 nm). The sections were stained with uranyl acetate (20 min) and lead acetate (20 min) and examined at 80 kV using an SEO TEM 100 (Sumy Electron Optics Transmission Electron Microscope 100, Ukraine) at Al-Azhar University in Cairo, Egypt, with a magnification power of x10,000^20^.

### Docking studies

**Fig. 2:**
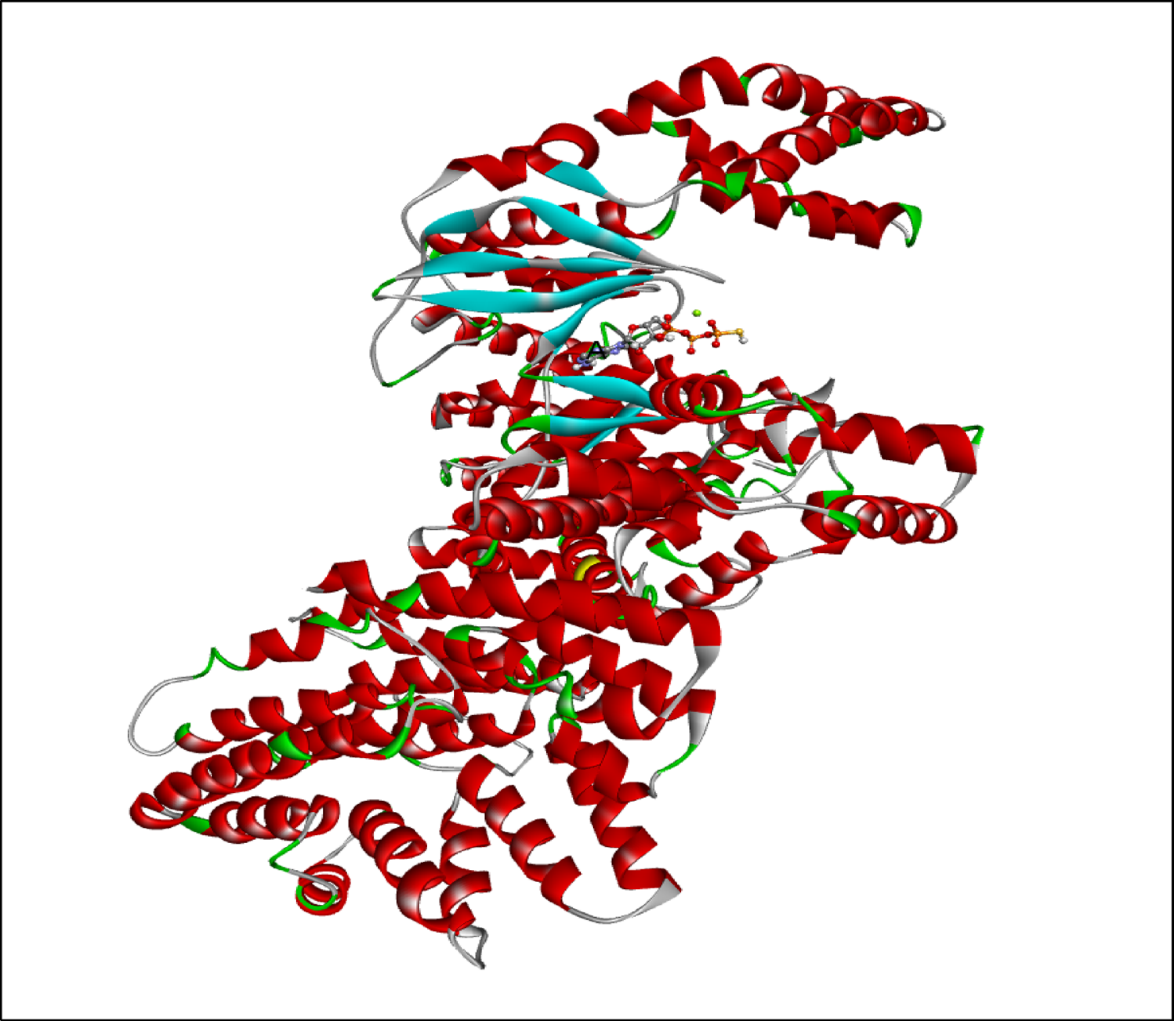
Structure of mTOR^ΔN^ ATP-γS-Mg complex. KD, kinase domain. ATP-γS-Mg complex is shown as ball and stick.

**Fig. 3:**
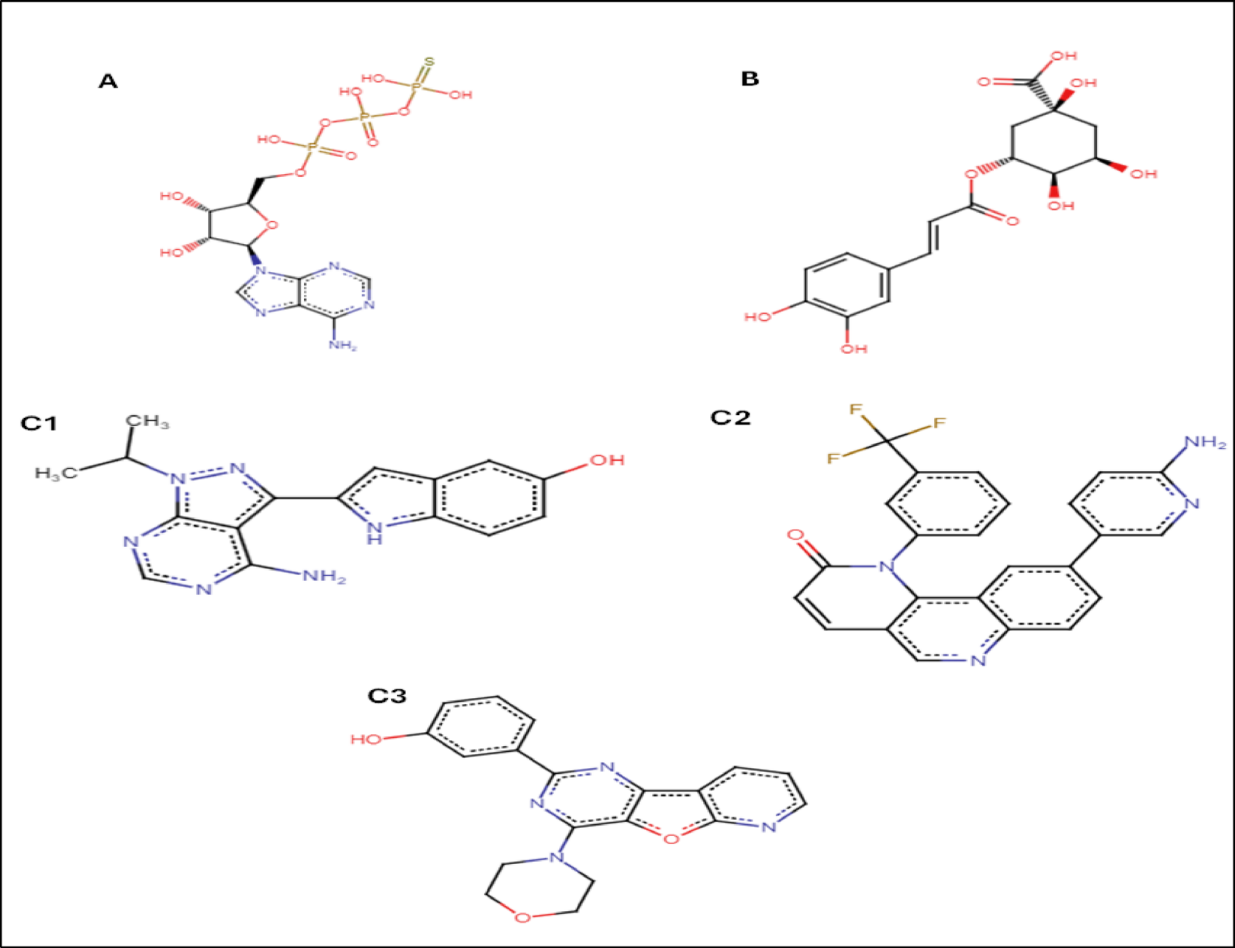
Ligands used in molecular docking. (**A**) ATP-gamma S co-crystallized ligand. (**B**) Chlorogenic acid. (**C**) Some ATP competitive inhibitors that were co-crystallized with mTOR protein, P242 (c1), Torin2 (c2) and PI-103 (c3) respectively.

AutoDock Vina modeling simulation software (AutoDock Vina v.1.2.5) was used to predict the protein-ligand binding affinity, as well as the preferred orientation of the docking pose between the amino acid residues that form the ATP-binding site of the mTOR^ΔN^ protein (N-terminally truncated human mTOR, residues 1376–2549; PDB: 4jsp) (Fig. 2) and chlorogenic acid, in addition to the co-crystallized ligands; ATP-γS-Mg complex (PDB: 4jsp), PP242 (2-[4-amino-1-(propan-2-yl)-1 H-pyrazolo[3,4-d]pyrimidin-3-yl]-1 H-indol-5-ol, PDB: 4jt5), Torin-2 (9-(6-aminopyridin-3-yl)-1-[3 (trifluoromethyl)phenyl]benzo[h][1,6]naphthyridin-2(1 H)-one, PDB: 4jsx) and PI-103 (3-(4-Morpholin-4-ylpyrido[3’,2’:4,5]furo[3,2-D]pyrimidin-2-yl)phenol, PDB: 4jt6) that were used as reference ligands (Fig. 3).

PyMOL molecular visualization tool (PyMOL v.2.5.4) (Schrödinger, Inc.) was used to extract the mTOR protein from its co-crystallized ligands, after adding hydrogen bonds to both. The extracted files were in the PDB format^21^. Auto-Dock (MGL-tools) was used to determine the docking site and the grid box dimensions of ligand binding pocket^22^. The grid box dimensions were selected by centering grid box on the ATP-gamma S-Mg complex, included in the crystal structure. Moreover, the target protein and the tested ligands were exported in PDBQT format (AutoDock format) using Open Babel v.2.3.1^23^.

A maximum of 9 poses was considered for each molecule where the target protein was kept as the rigid receptor while keeping the conformation of the ligands as flexible^24^. Finally, the most favorable pose was selected according to the minimum free energy of the protein–ligand complex and for visualizing the type of interactions between the ligand and the protein, BIOVIA Discovery Studio (DS) Visualizer v.4.5. was used.

### Statistical analysis

SPSS version 24 for Windows was used for all data analysis. Data were compared using one-way analysis of variance (ANOVA), followed by a least significant difference post hoc multiple comparison test. The data were represented as mean ± SD and were considered statistically significant when the *P* value was ≤ 0.05.

## Results

### Identification of active components of *Arabica coffee* extract

**Fig. 4:**
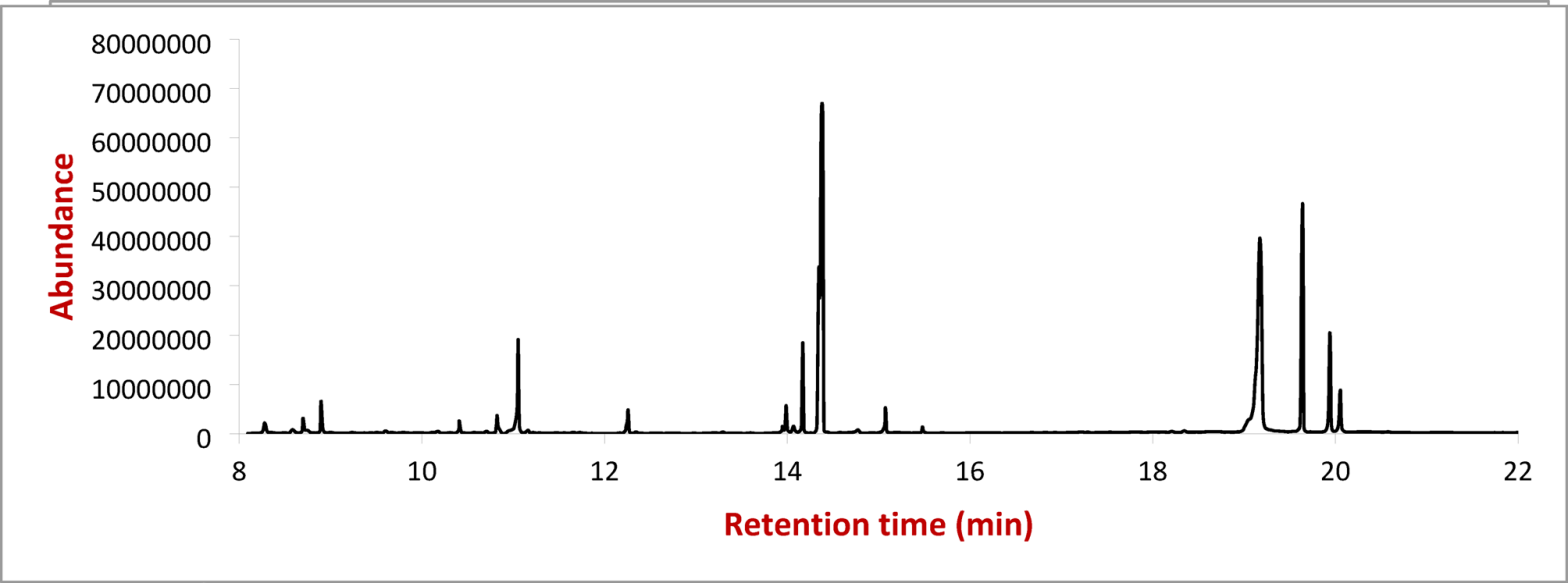
GC/MS chromatogram of the water extract of *Arabica coffee.*

**Fig. 5:**
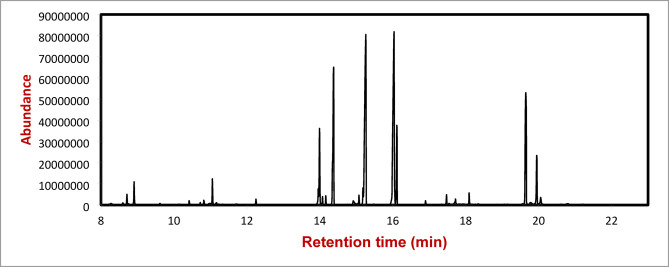
GC/MS chromatogram of ethanolic extract of *Arabica coffee*.

**Fig. 6:**
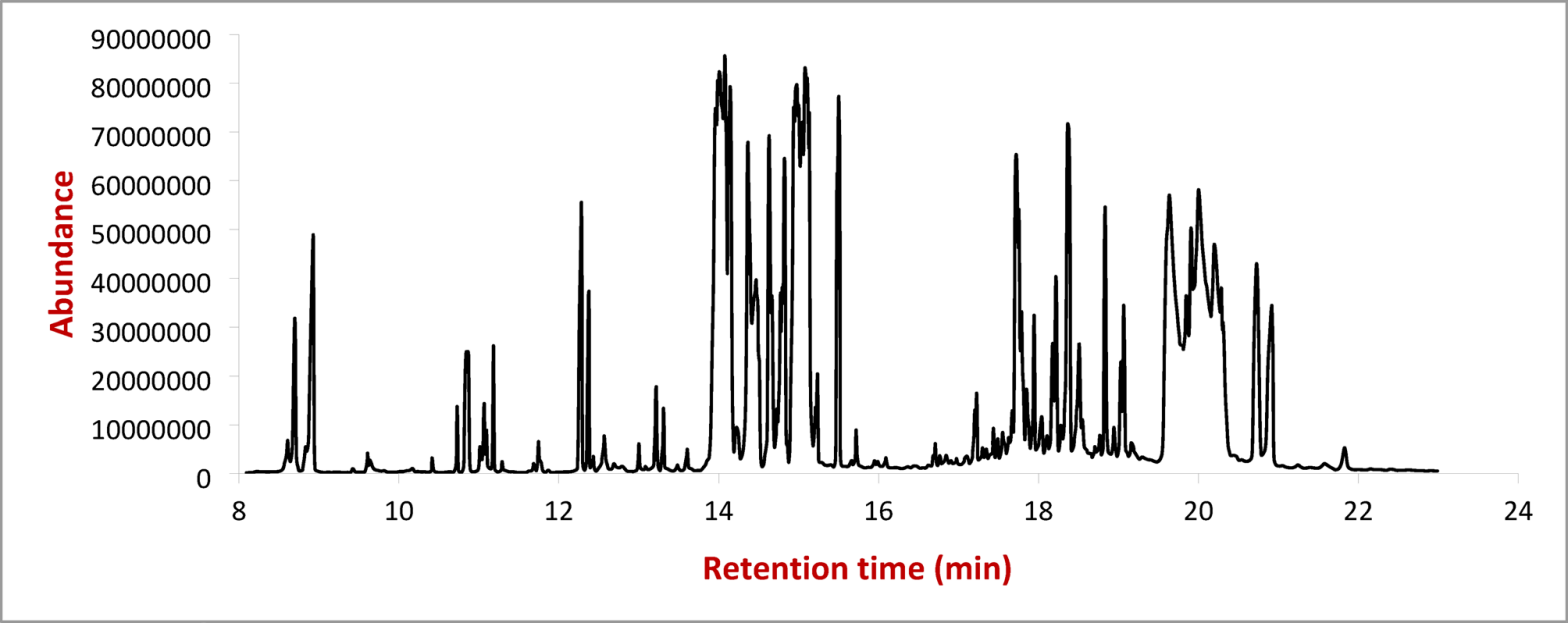
GC/MS chromatogram of the methanolic extract of *Arabica coffee.*

The phytochemical analysis of *Arabica coffee* extract by GC/MS (Tables 1, 2 and 3) revealed that a higher number of compounds were extracted from the methanol compared with water or ethanol extraction. The methanolic extract contains the highest percentage of chlorogenic acid (12.7963%), compared with aqueous and ethanolic extracts were 12.3472 and 12.0889%, respectively (Figs. 4, 5 and 6).

**Table 1:** Phytochemical analysis of bioactive compounds in the water extract of *Arabica coffee* by GC/MS.

Peak	Retention time	Area percentage	Name of compounds
1	8.284	0.905	Tris(trimethylsilyl)carbamate
2	8.5962	0.3939	Thieno[2,3-b]pyridin-3-amine, 4,6-dimethyl-2-phenylsulfonyl-
3	8.7049	0.8909	Ethylene glycol, 2TMS derivative
4	8.9017	1.3989	Glycolic acid, 2TMS derivative
5	9.6145	0.2729	Glycolic acid, 2TMS derivative
6	9.825	0.1589	Pentasiloxane, dodecamethyl-
7	10.178	0.2378	Pentasiloxane, dodecamethyl-
8	10.4156	0.6096	4-Hydroxybutanoic acid, 2TMS derivative
9	10.7211	0.2391	L-(+)-Threose, tris(trimethylsilyl) ether, ethyloxime (isomer 1)
10	10.8297	1.0271	Silanol, trimethyl-, phosphate (3:1)
11	11.0537	4.325	Butanedioic acid, 2TMS derivative
12	11.1691	0.1403	Glyceric acid, 3TMS derivative
13	11.2302	0.1362	2-Tributylsilyloxypentane
14	11.6715	0.228	.alpha.-Ketoisovaleric acid, TMS derivative
15	12.2553	1.2851	Malic acid, 3TMS derivative
16	12.3504	0.1659	2-Butenedioic acid, (Z)-, 2TBDMS derivative
17	13.2125	0.1839	L-(+)-Threose, tris(trimethylsilyl) ether, trimethylsilyloxime (isomer 1)
18	13.3008	0.1135	D-(+)-Ribono-1,4-lactone, 3TMS derivative
19	13.9932	1.7874	D-Arabino-Hexonic acid, 3-deoxy-2,5,6-tris-O-(trimethylsilyl)-, .gamma.-lactone
20	14.0747	0.4943	9(1 H)-Phenanthrone, 2,3,4,4a,4b,5,6,7,8,8a-decahydro-
21	14.1697	4.5391	Citric acid, 4TMS derivative
22	14.3802	27.041	Quininic acid (5TMS)
23	14.7807	0.5722	Cyanuric acid, 3TMS derivative
24	14.9232	0.1472	Myo-Inositol, 6TMS derivative
25	15.0794	1.1856	Scyllo-Inositol, 6TMS derivative
26	15.2152	0.2252	Palmitic Acid, TMS derivative
27	15.4867	0.3298	Myo-Inositol, 6TMS derivative
28	16.0909	0.1519	.beta.-D-Galactopyranoside, methyl 2,3-bis-O-(trimethylsilyl)-, cyclic methylboronate
29	18.209	0.2377	3 H-pyrazol-3-one, 2,4-dihydro-5-(methylthio)-2-(2,4,6-trichlorophenyl)-
30	18.3515	0.116	D-(+)-Galacturonic acid, 5TMS derivative
31	19.1729	24.0233	Lactose, 8TMS derivative
32	19.6413	12.3472	Chlorogenic acid (6TMS)
33	19.94	5.8806	2-Morpholino-2-phenyl-1,3-indandione
34	20.0555	2.2093	Quininic acid (5TMS)

**Table 2:** Phytochemical analysis of bioactive compounds in the ethanolic extract of *Arabica coffee* by GC/MS.

Peak	Retention time	Area percentage	Name of compounds
1	8.284	0.4012	Tris(trimethylsilyl)carbamate
2	8.6098	0.2877	Thieno[2,3-b]pyridin-3-amine, 4,6-dimethyl-2-phenylsulfonyl-
3	8.7116	0.804	Lactic Acid, 2TMS derivative
4	8.9085	1.3148	Glycolic acid, 2TMS derivative
5	9.6213	0.0869	2-Propenoic acid, 2-[(trimethylsilyl)oxy]-, trimethylsilyl ester
6	10.178	0.1697	1,1,1,3,5,5,7,7,7-Nonamethyl-3-(trimethylsiloxy)tetrasiloxane
7	10.4224	0.265	4-Hydroxybutanoic acid, 2TMS derivative
8	10.7279	0.2077	D-(-)-Erythrose, tris(trimethylsilyl) ether, methyloxime (anti)
9	10.8229	0.4018	Silanol, trimethyl-, phosphate (3:1)
10	11.0605	1.9898	Butanedioic acid, 2TMS derivative
11	11.1691	0.187	Glyceric acid, 3TMS derivative
12	11.237	0.2019	Butyramide, 3-(2-furyl)-N-phenyl-
13	11.3456	0.2432	Butanedioic acid, 2TMS derivative
14	11.6783	0.3323	.alpha.-Ketoisovaleric acid, TMS derivative
15	12.072	0.1526	4-Methoxybenzeneacetic acid, TMS derivative
16	12.2553	0.4321	Malic acid, 3TMS derivative
17	12.3504	0.1315	Tartronic acid, 3TMS derivative
18	13.9932	6.0121	D-Arabino-Hexonic acid, 3-deoxy-2,5,6-tris-O-(trimethylsilyl)-, .gamma.-lactone
19	14.0747	0.4654	D-2-Deoxyribose, 3TMS derivative
20	14.1697	0.4701	Citric acid, 4TMS derivative
21	14.3734	12.6954	Quininic acid (5TMS)
22	14.7875	0.1446	Glutaconic acid, tris(trimethylsilyl)-(ester)
23	14.93	0.7099	Caffeine
24	15.0794	0.6809	Myo-Inositol, 6TMS derivative
25	15.2559	23.6316	Palmitic Acid, TMS derivative
26	15.4867	0.086	Acrylic acid, 2,3-bis[(trimethylsilyl)oxy]-, trimethylsilyl ester
27	16.0298	22.2984	9,12-Octadecadienoic acid (Z, Z)-, TMS derivative
28	16.1112	5.2593	Stearic acid, TMS derivative
29	16.9055	0.2572	Arachidic acid, TMS derivative
30	17.4758	0.5822	1-Monopalmitin, 2TMS derivative
31	17.5504	0.1479	3,10-Dioxa-2,11-disiladodeca-5,7-diene, 2,2,11,11-tetramethyl-
32	17.7202	0.6071	D-(+)-Cellobiose, octakis(trimethylsilyl) ether, methyloxime (isomer 2)
33	18.1003	0.7992	1-Monooleoylglycerol, 2TMS derivative
34	18.3515	0.061	Lactulose, octakis(trimethylsilyl) ether, methyloxime (isomer 2)
35	19.6481	12.0889	Chlorogenic acid (6TMS)
36	19.7907	0.4484	Ginkgolide B 3TMS
37	19.9468	3.7859	Hypoxanthine, 2TBDMS derivative
38	20.0622	0.8184	2-(Hydroxyimino)-N-(2-iodophenyl)acetamide, 2TMS derivative
39	20.8022	0.2293	2-Ethoxyheptylphthalimide
40	21.2163	0.1115	.beta.-Sitosterol, TMS derivative

**Table 3:** Phytochemical analysis of bioactive compounds in the methanolic extract of *Arabica coffee* by GC/MS.

Peak	Retention time	Area percentage	Name of compounds
1	8.2297	0.0231	4-Phenyl-2-butanol, TBDMS derivative
2	8.6098	0.2600	Tartronic acid, 3TMS derivative
3	8.6981	0.8633	Lactic Acid, 2TMS derivative
4	8.9221	1.8822	Glycolic acid, 2TMS derivative
5	9.4313	0.0177	3-Hydroxyisovaleric acid, 2TMS derivative
6	9.6145	0.1644	Hydracrylic acid, 2TMS derivative
7	9.8182	0.0107	1,3-Butanediol, TBDMS derivative
8	10.178	0.0527	Pentasiloxane, dodecamethyl-
9	10.4224	0.0509	4-Hydroxybutanoic acid, 2TMS derivative
10	10.7347	0.1877	Glycerol, 3TMS derivative
11	10.8569	1.0743	Silanol, trimethyl-, phosphate (3:1)
12	11.0673	0.5166	Butanedioic acid, 2TMS derivative
13	11.1827	0.3550	Glyceric acid, 3TMS derivative
14	11.2913	0.0376	2-Pentenoic acid, 2-[(trimethylsilyl)oxy]-, trimethylsilyl ester
15	11.3592	0.019	2-Butyne-1,4-diol, 2TMS derivative
16	11.753	0.2219	5-Amino-1-tetrazolylacetic acid, 2TMS derivative
17	11.8752	0.0117	9(1 H)-Phenanthrone, 2,3,4,4a,4b,5,6,7,8,8a-decahydro-
18	12.0856	0.0191	2-Hydroxyphenethyl alcohol, 2TMS derivative
19	12.2757	1.5020	Malic acid, 3TMS derivative
20	12.3707	0.7616	2,2-Dimethylpropane-1,3-diol, O,O’-bis(trimethylsilyl)-
21	12.5744	0.2735	4-Trimethylsiloxy(trimethylsilyl)valerate
22	12.7034	0.0981	Methylmalonic acid, 2TMS derivative
23	12.7848	0.0798	Silane, [[3,3-dimethyl-4-methylene-2-(trimethylsilyl)-1-cyclopenten-1-yl]methoxy]trimethyl-
24	13.0021	0.113	D-(-)-Ribofuranose, tetrakis(trimethylsilyl) ether (isomer 2)
25	13.0971	0.0693	.beta.-D-(+)-Xylopyranose, 4TMS derivative
26	13.2193	0.3725	Glyoxime, 2TMS derivative
27	13.3143	0.2691	2-Butenedioic acid, (E)-, 2TMS derivative
28	13.4841	0.0538	9(1 H)-Phenanthrone, 2,3,4,4a,4b,5,6,7,8,8a-decahydro-
29	13.6063	0.1624	Levoglucosan, 3TMS derivative
30	14.0136	7.5284	D-Arabino-Hexonic acid, 3-deoxy-2,5,6-tris-O-(trimethylsilyl)-, .gamma.-lactone
31	14.1426	3.0829	D-Arabino-Hexonic acid, 3-deoxy-2,5,6-tris-O-(trimethylsilyl)-, .gamma.-lactone
32	14.2308	0.4378	Gulonic acid, .gamma.-lactone, 4TMS derivavative
33	14.3734	3.0427	Quininic acid (5TMS)
34	14.4684	2.4687	Quininic acid (5TMS)
35	14.6381	2.627	Molybdenum, tricarbonyl[(1,2,3,4,5,6-.eta.)-1,4-dimethylbenzene]-
36	14.8214	3.0678	Cyanuric acid, 3TMS derivative
37	14.9708	7.9106	Caffeine
38	15.093	4.1263	Iron, dicarbonyl(.eta.5 − 2,4-cyclopentadien-1-yl)(trimethylsilyl)-
39	15.2355	0.6846	Palmitic Acid, TMS derivative
40	15.5003	2.5657	Myo-Inositol, 6TMS derivative
41	15.7243	0.3406	Caffeic acid, 3TMS derivative
42	15.9551	0.1862	D-Xylose, 4TMS derivative
43	16.0977	0.1138	7,10,13,16-Docosatetraenoic acid, (Z)-, TMS derivative
44	16.1792	0.0485	5.alpha.-Pregnan-3.beta.,20.beta.-diol
45	16.2538	0.0361	Pentanedioic acid, 3-oxo-, tris(trimethylsilyl) ester
46	16.3692	0.0728	Allonic acid, .gamma.-lactone, 4TMS derivavative
47	16.4371	0.0822	Glyceric acid, 3TMS derivative
48	16.7087	0.2627	.beta.-D-Glucopyranuronic acid, 5TMS derivative
49	16.7698	0.092	.alpha.-D-Glucopyranuronic acid, 5TMS derivative
50	16.8512	0.2367	.beta.-D-Galactofuranoside, ethyl 2,3,5,6-tetrakis-O-(trimethylsilyl)-
51	16.9802	0.0911	5-Methyluridine, 3TMS derivative
52	17.1092	0.2102	(-)-Globulol
53	17.2246	0.553	L-(-)-Sorbofuranose, pentakis(trimethylsilyl) ether
54	17.3061	0.286	D-Psicofuranose, pentakis(trimethylsilyl) ether (isomer 2)
55	17.4418	0.4760	.alpha.-D-Galactofuranose, 1,2,3,5,6-pentakis-O-(trimethylsilyl)-
56	17.5573	0.3354	D-Psicofuranose, pentakis(trimethylsilyl) ether (isomer 2)
57	17.727	4.9296	Sucrose, 8TMS derivative
58	17.9442	0.7278	3-.alpha.-Mannobiose, octakis(trimethylsilyl) ether (isomer 2)
59	18.0392	0.4722	3,4-Heptadien-2-one, 3,5-dicyclopentyl-6-methyl-
60	18.1071	0.2638	D-(-)-Lyxose, tetrakis(trimethylsilyl) ether, methyloxime (syn)
61	18.2225	1.6739	Quininic acid (5TMS)
62	18.3787	3.2936	.beta.-Lactose, 8TMS derivative
63	18.5077	1.6405	D-(+)-Galacturonic acid, 5TMS derivative
64	18.7656	0.3813	6-(3,5-Dichlorobenzoyl)-7-methyl-6,7-dihydro-5 H-pyrrolo[3,4-d]pyrimidine-2,4-diamine, 2TMS derivative
65	18.8335	1.3799	Galactopyranose, 5TMS derivative
66	18.9489	0.2961	1-Hexene, 1-(9-borabicyclo[3.3.1]non-9-yl)-2-(9-borabicyclo[3.3.1]non-9-ylthio)-
67	19.0711	1.3600	Galactinol, nonakis(trimethylsilyl) ether
68	19.173	0.4116	3-Bromo-N-(3,5-dichlorophenyl)benzamide, TMS derivative
69	19.3087	0.3721	Cortisone
70	19.6414	12.7963	Chlorogenic acid (6TMS)
71	20.008	8.7054	Quinoline, 6-bromo-2-trifluoromethyl-4-methoxy-
72	20.7276	2.3845	2-Morpholino-2-phenyl-1,3-indandione
73	20.9108	1.9264	Quinoline, 6-bromo-2-trifluoromethyl-4-methoxy-
74	21.2435	0.1011	D-(-)-Ribose, tetrakis(trimethylsilyl) ether, benzyloxime (isomer 2)
75	21.5829	0.1512	3,4-Heptadien-2-one, 3,5-dicyclopentyl-6-methyl-
76	21.8273	0.2305	D-(-)-Lyxofuranose, tetrakis(trimethylsilyl) ether
77	22.4043	0.0135	3-.alpha.-Mannobiose, octakis(trimethylsilyl) ether (isomer 2)

### The autophagy markers analysis

**Fig. 7:**
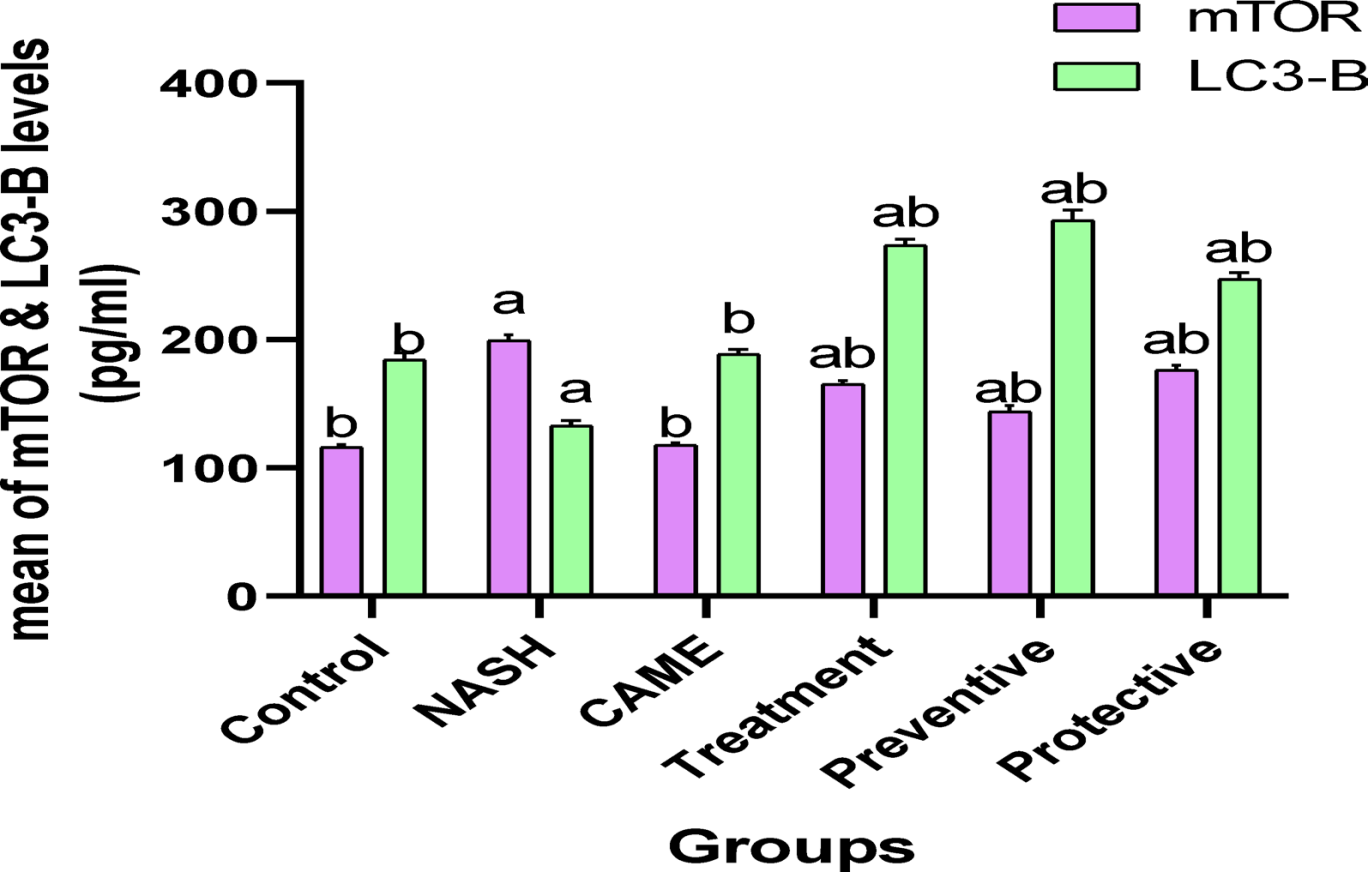
Mean of mTOR & LC3-B levels in liver tissue of the different groups where (a) significance vs. control group, (b) significance vs. NASH group.

The obtained data showed that mTOR level in liver tissue was increased significantly (*p* ≤ 0.001) in the NASH group while the LC3-B level was significantly decreased (*P* ≤ 0.001) compared to the control group. However, rats treated with CAME revealed a significant reduction (*p* ≤ 0.001) in the mTOR level and a significant increase (*P* ≤ 0.001) in LC3-B level in comparison with the NASH group. In addition, the treated, preventive, and protective groups demonstrated a significant increase (*P* ≤ 0.001) in LC3-B level compared to the untreated group. There was no significant difference in the level of mTOR and LC3-B in the CAME group versus the control group (Table 4) & (Fig. 7).

**Table 4:** Statistical analysis (ANOVA) for mTOR and LC3-B in liver tissue in the different groups.

Groups Parameters	Group (1) Control	Group (2) NASH	Group (3) CAME	Group (4) Treatment	Group (5) Preventive	Group (6) Protective
mTOR (pg/mL)	116.33 ± 1.73^b^	199.65 ± 4.3^a^	117.73 ± 1.7^b^	165.48 ± 2.74^ab^	143.94 ± 4.72^ab^	176.39 ± 3.92^ab^
LC3-B (pg/mL)	184.53 ± 5.16^b^	132.71 ± 4.22^a^	188.72 ± 3.92^b^	273.66 ± 4.47^ab^	293.14 ± 8.19^ab^	247.31 ± 4.7^ab^

Each value is represented as mean ± standard deviation (mean ± SD) (*N* = 6 animals per group). Data with different superscripts are significantly different at *p* ≤ 0.05.

a significance vs. control group, b significance vs. NASH group.

### Pathological alterations examination by transmission electron microscope

**Fig. 8:**
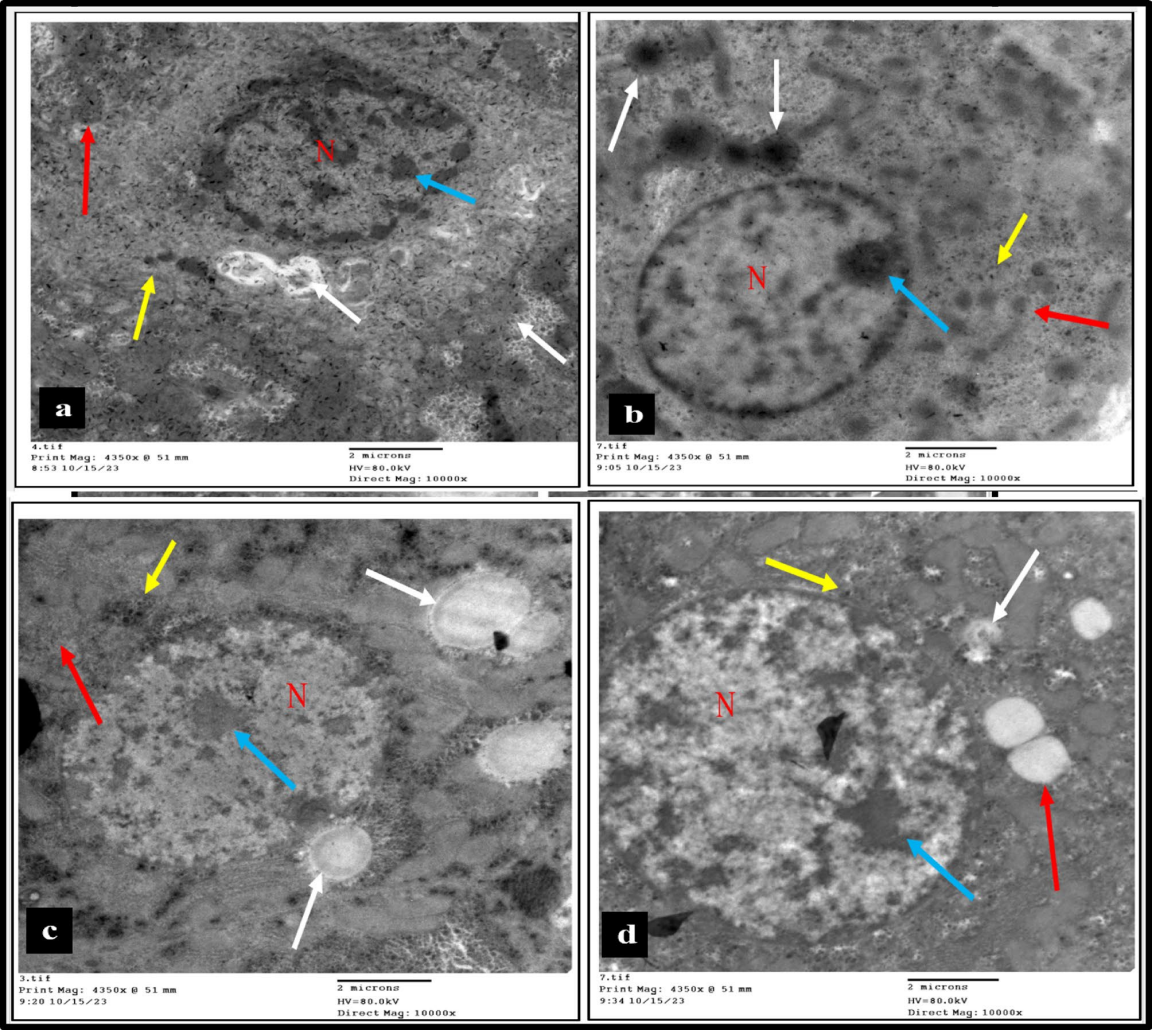
(**a**) NASH group: liver manifested pyknotic nucleus (N) with clumped chromatin (blue arrow), average mitochondria (red arrow), many cytoplasmic vacuoles (white arrow), and electron dense bodies (yellow arrow) (10000 x). (**b**) Treatment group: liver expressed nucleus (N) with prominent nucleolus (blue arrow), scattered small mitochondria (red arrow), many large autophagosomes with double layers (white arrow), and few electron dense bodies (yellow arrow) (10000 x). (**c**) Preventive group: liver displayed nucleus (N) with prominent nucleolus (blue arrow), average ovoid mitochondria (red arrow), many large autophagosomes with double layers (white arrow), and few electron dense bodies (yellow arrow) (10000 x). (**d**) Protective group: liver unveiled nucleus (N) with prominent nucleoli (blue arrow), many swollen mitochondria (red arrow), few small autophagosomes with double layers (white arrow), and few small electron dense bodies (yellow arrow) (10000 x).

Histological examination of liver tissue by electron microscope illustrated that the liver of the NASH group revealed small pyknotic nuclei with clumped chromatin, scattered small, condensed mitochondria, many small autophagosomes with double layers, many cytoplasmic vacuoles, many electron-dense bodies, and mildly congested blood vessels, as shown in Fig. 8a. On the other hand, the liver of the treatment group manifested average nuclei with prominent nucleoli and dispersed chromatin, scattered small mitochondria, many large autophagosomes with double layers, few electron-dense bodies, and mildly congested blood vessels, as shown in Fig. 8b. In the prevented group, the liver exhibited average nuclei with prominent nucleoli and dispersed chromatin, scattered swollen mitochondria, many large autophagosomes with double layers, a few small and large cytoplasmic vacuoles, a few small and large electron dense bodies, and an average endoplasmic reticulum, as shown in Fig. 8c. While the protected group demonstrated a liver with average nuclei with prominent nucleoli and clumped chromatin, many swollen mitochondria, a few small autophagosomes with double layers, a few small cytoplasmic vacuoles, and a few small electron dense bodies, as shown in Fig. 8d.

### Docking studies

The docking results, presented as binding free energy and the interacting ligand binding pocket residues, are tabulated in (Table 5). Chlorogenic acid exhibited binding energy − 7.554 which reveals that the binding mode of chlorogenic acid is more favorable than that of ATP-γS and intermediate compared with that of other inhibitors. The structure of chlorogenic acid is characterized by the presence of many phenolic (as in P242 and PI-103) and non-phenolic hydroxyl groups that can act as hydrogen donors and acceptors in the formation of many hydrogen bonds with the hydrophilic side chain of Asp 2357 (that serves as metal ligand in the catalytic cleft) Lys 2187 and Gly 2238.

**Table 5:** The binding free energies (γG) and binding site residues of interaction.

Ligands	ΔG (kcal/mol)	Interacted ligand binding pocket residues
ATP-γS-Mg complex	-6.563	Leu 2185, Gly 2238, Trp 2239, Val 2240, Met 2345, Ile 2356
PP242	-7.151	Pro 2169, Leu 2185, Lys 2187, Trp 2239, Met 2345, Ile 2356
Torin-2	-9.982	Ile 2163, Pro 2169, Leu 2185, Lys 2187, Tyr 2225, Ile 2237, Trp 2239, Cys 2243, Met 2345, Ile 2356
PI-103	-8.282	Pro 2169, Leu 2185, Trp 2239, Val 2240, Thr 2245, Ala 2248
Chlorogenic acid	-7.554	Leu 2185, Lys 2187, Gly 2238, Trp 2239, Val 2240, Met 2345, Asp 2357

**Fig. 9:**
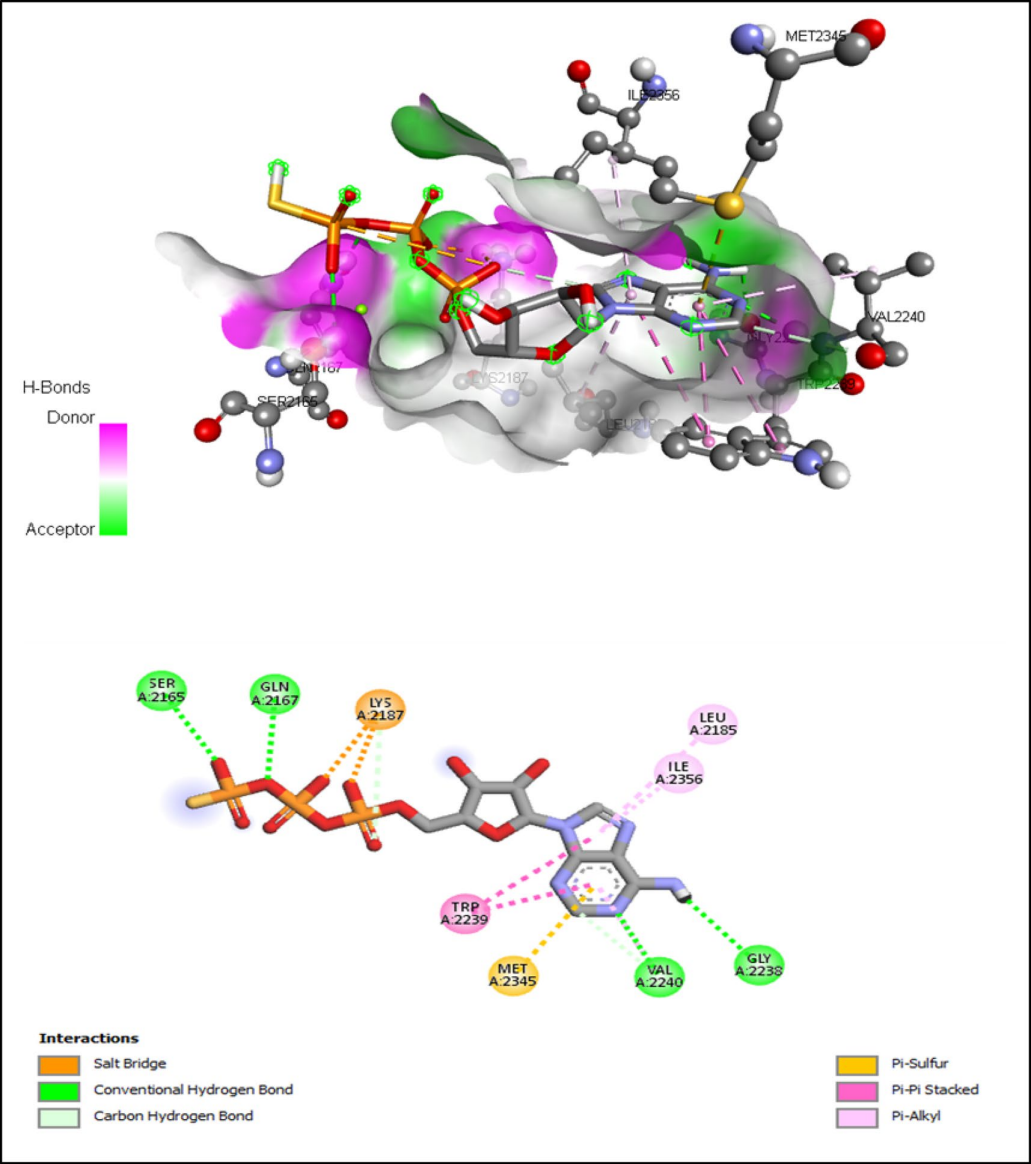
Interactions of ATP-binding pocket residues with ATP-γS-Mg complex (3D (up) and 2D (down)).

**Fig. 10:**
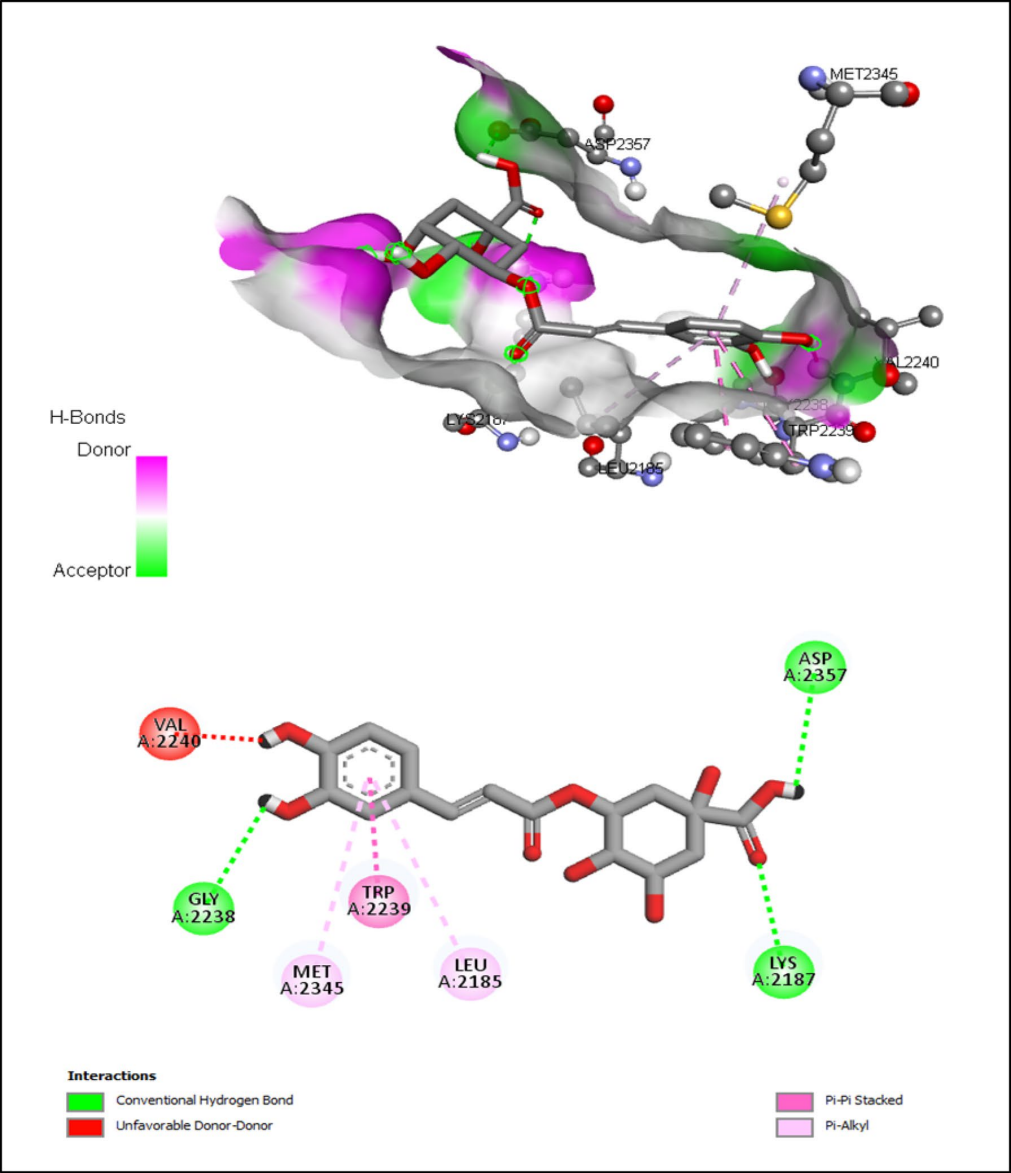
Interactions of ATP-binding pocket residues with chlorogenic acid (3D (up) and 2D (down)).

Compared to the binding mode of chlorogenic acid and ATP-γS (Fig. 9), we found that the benzene ring of chlorogenic acid binds to the adenine pocket and form extensive hydrophobic stacking interactions with non-polar amino acid residues as the indole group of Trp 2239 and hydrophobic side chains of Met 2345 and Leu 2185, as shown in Fig. 10.

**Fig. 11:**
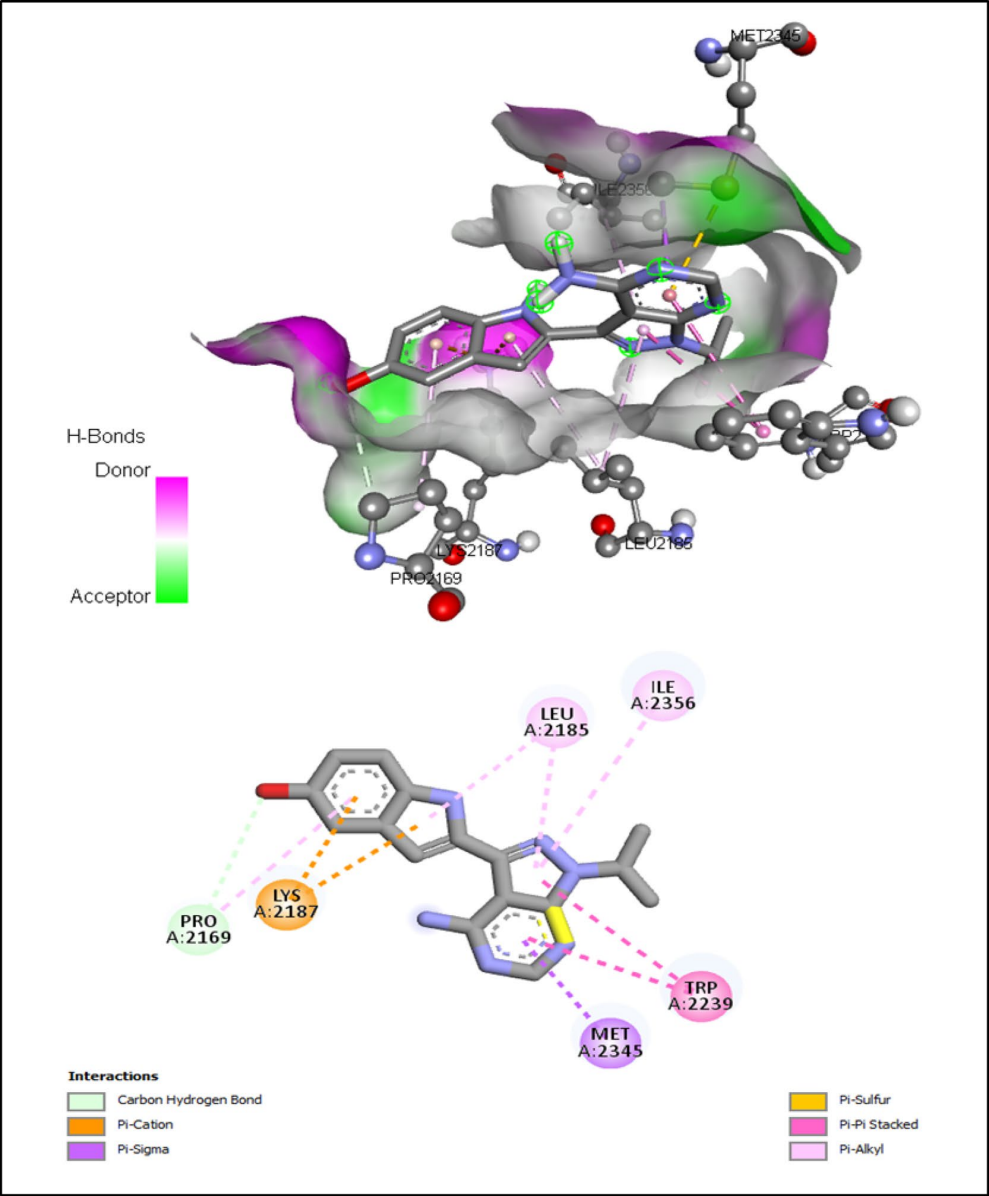
Interactions of ATP-binding pocket residues with PP242 (3D (up) and 2D (down)).

**Fig. 12:**
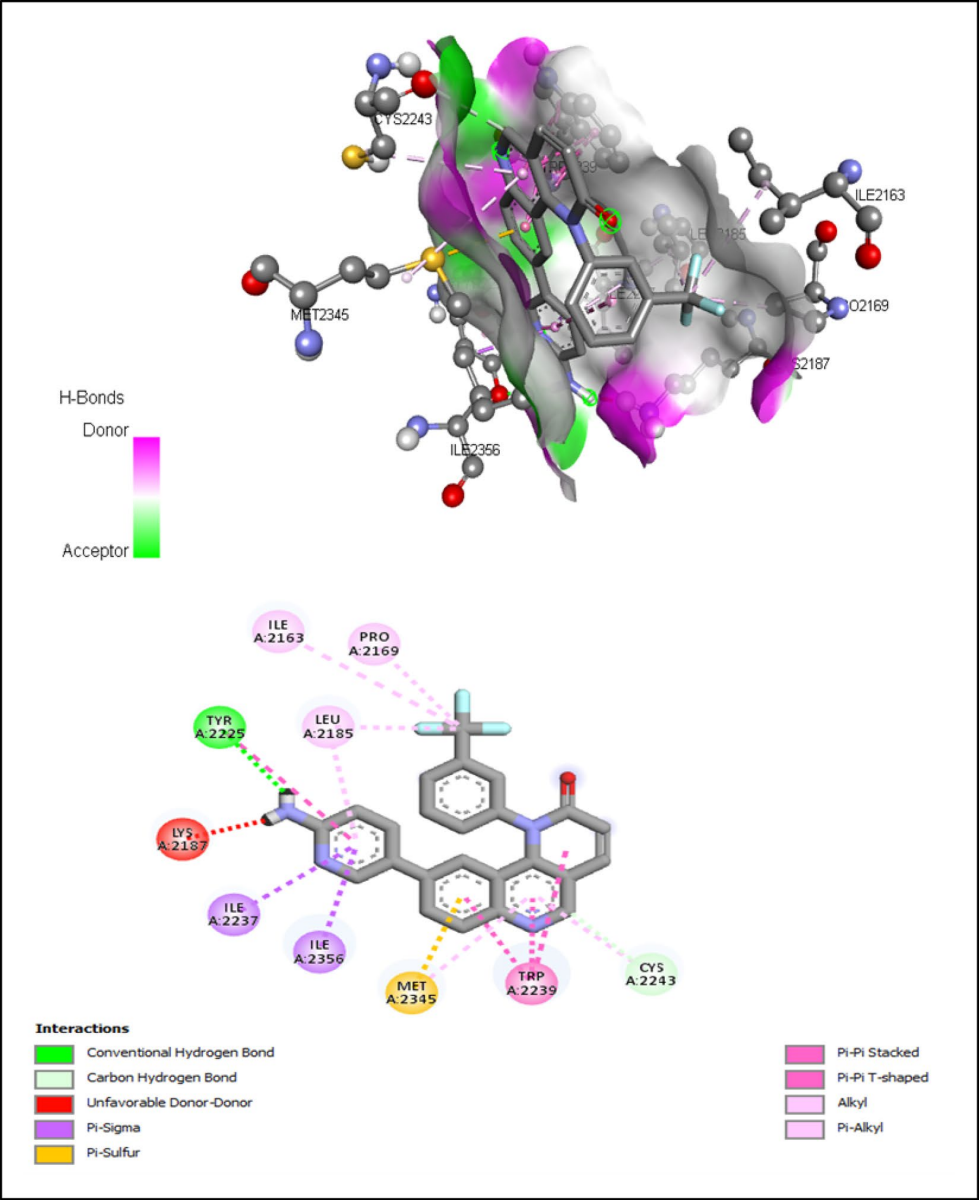
Interactions of ATP-binding pocket residues with Torin-2 (3D (up) and 2D (down)).

**Fig. 13:**
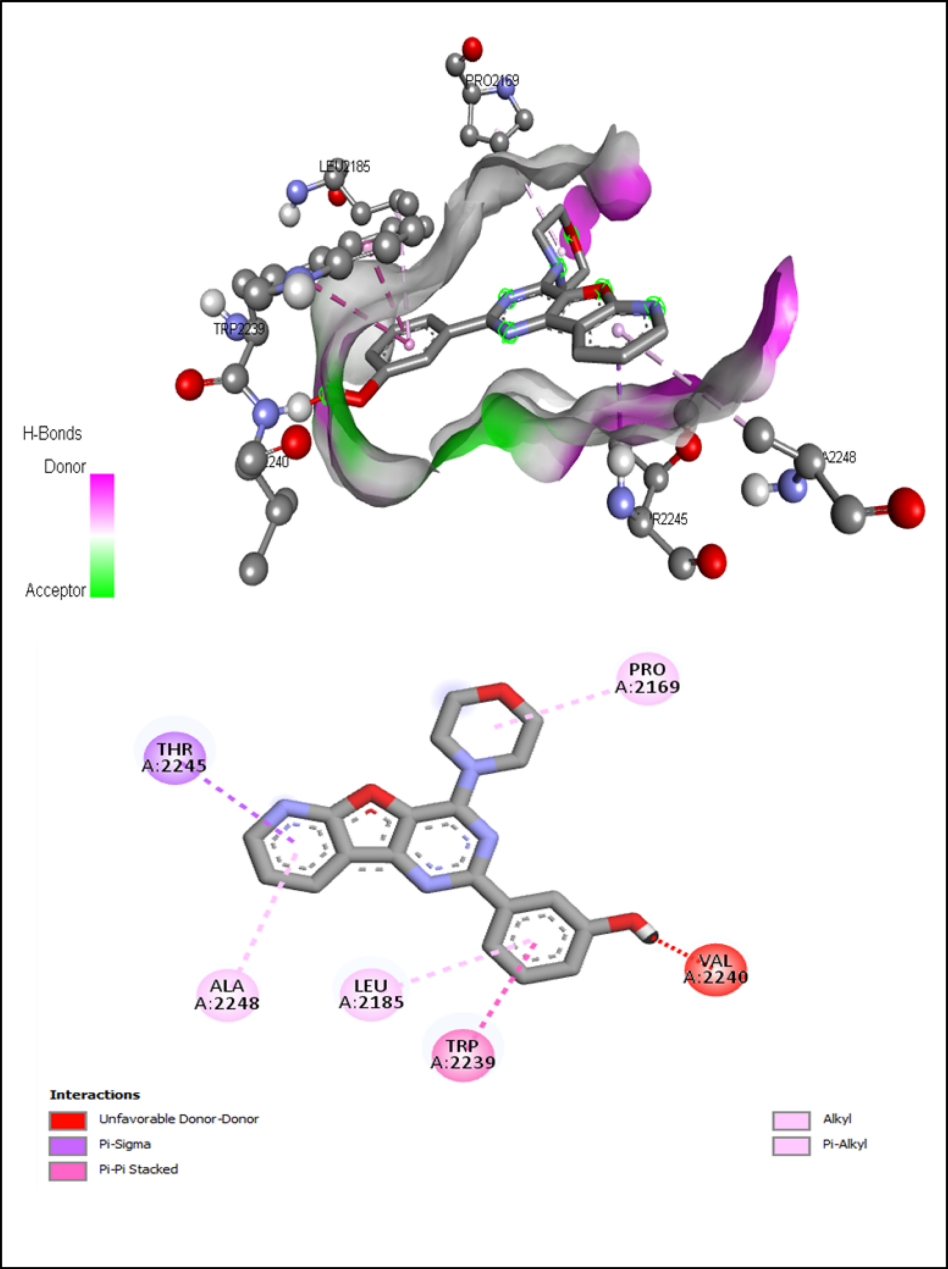
Interactions of ATP-binding pocket residues with PI-103 (3D (up) and 2D (down)).

The interactions of ATP-binding pocket residues with PP242, Torin-2, and PI-103 were shown in Figs. 11 and 12, and 13, respectively.

## Discussion

Nonalcoholic steatohepatitis (NASH), an inflammatory subtype of nonalcoholic fatty liver disease (NAFLD). It is linked to the advancement of fibrosis and cirrhosis. NASH is becoming a more common chronic liver disease^25^. Treatment options for NASH include dietary and lifestyle changes, weight loss, insulin sensitization, and lipid-lowering medications to prevent or treat this condition^26^. Some drugs as antiarrhythmic agents, chemotherapeutics, and TET can cause steatosis as their site effects, which inhibits the action of enzymes that are involved in the release of lipoproteins from the liver^27^. The most common side effects of prolonged and high doses of TET were steatosis and liver damage in animal models^2^. Autophagy has been implicated in the pathophysiology of NAFLD, as it has been discovered that this route promotes the breakdown of intracellular lipids in hepatocytes and may therefore regulate the development of hepatic steatosis^28^.

The development of NAFLD and lipid imbalance have been linked to autophagy, according to certain theories^29^. The efficacy of natural products as curcumin, bergamot, and resveratrol, in improving NASH has been demonstrated by different studies^30^. Herbal medicine extracts which induce autophagy may play a role in the therapy of NASH^31^.

Microtubule-Associated Protein 1 Light Chain 3 Beta, or LC3B, is an essential protein in autophagy that serves as both a marker and a facilitator. It facilitates the union of lysosomes and autophagosomes to produce the autolysosome, which is where degradation takes place^9^. Moreover, beyond its membrane functions, LC3B binds to particular mRNA sequences (such AAUAAA) and causes their quick destruction (LC3B-mediated mRNA decaying), which aids in the removal of autophagy’s negative regulators like PRMT1 and advances the process^32^.

While autophagy markers such as LC3-II and p62/SQSTM1 show the activation and flux of autophagy, mTOR is a master regulator that inhibits autophagy; mTOR suppression results in declined LC3-II transformation and p62 buildup, whereas suppression of mTOR boosts autophagy, via boosting LC3-II and degrading p62, highlighting their inverse relationship^33,34^.

Activated mTORC1 primarily phosphorylates ULK1/2 and the VPS34 complex to prevent the induction of autophagy^34^. In the present study. There was a remarkable elevation in mTOR level with a decrease in LC3-B level in TET injected animals, which indicates that TET can cause liver tissue injury. These findings were in agreement with He et al.^35^ who elucidated that, Beclin1 and LC3, two autophagy-related proteins, exhibit markedly reduced levels of both mRNA and protein in the NAFLD rat model.

Additionally, Nakadera et al.^36^ reported that mTOR can be used as an indication to assess autophagic dysfunction in NAFLD since it regulates both lysosomal and autophagic acidification via modulating the expression of V-ATPase. Also, González-Rodríguez et al.^37^ revealed that autophagic flux becomes impaired in the livers of mice models of NAFLD fed a high-fat diet, as well as in humans with NAFLD and NASH. It was noticed that an excess of fatty acids led to a notable rise in endoplasmic reticulum stress, inhibition of the autophagic flow, and programmed cell death.

Furthermore, Noureddin et al.^38^ stated that impaired autophagic function may facilitate the development and progression of hepatic steatosis towards liver damage. Furthermore, a tendency to NASH is influenced by defective autophagic functions in the liver. In both humans and mice, the incidence of NASH rises with age, and this could be due to a reduction in autophagic flux. Moreover, Yang et al.^39^ declared that obese mice fed a high-fat diet also exhibit diminished hepatic autophagic function, as evidenced by lower levels of Beclin-1 and LC3-II and fewer autophagosomes and autolysosomes.

In the current study, the rats administrated CAME resulted in an increase in LC3-B level, and a reduction in mTOR level compared with the NASH group. These results were agreed with Meng et al.^40^, who stated that chlorogenic acid induced autophagy in hepatocytes by elevating the ULK1 expression level and promoting LC3B-I transformation into LC3B-II in the liver tissues of mice with hepatic steatosis induced by high fat diet. Also, Yan et al.^41^ mentioned that in a rat model of NAFLD, chlorogenic acid reduces liver injury via triggering autophagy.

Moreover, Takahashi et al.^42^ proved that phytochemicals may enhance liver protection by regulating the autophagic response. It has been observed that drinking coffee lowers hepatic mTOR levels in old mice. Additionally, Saiki et al.^43^ observed that caffeine increases autophagic response by downregulating PI3K/Akt/mTOR/p70S6K signaling. However, Parafati et al.^44^ indicated that autophagy plays a protective role in NAFLD in which bergamot polyphenol fraction improves hepatic steatosis by activating autophagy via raising lC3-B and beclin-1 levels.

Also, natural products were used to explain the relationship between the mTOR pathway and LC3B levels, such as quercetin, a flavonoid compound with anti-oxidative properties that alleviated atherosclerosis lesions induced by a high-fat diet in ApoE/mice, which reduced lipid accumulation in aortic roots. Additionally, the ratio of LC3 II/I in mouse aortas was significantly elevated, while mTOR, P53, and P21 protein expression levels were down regulated^45^.

Also, nicotinate-curcumin, a compound synthesized from nicotinate and curcumin, has beneficial effects on the prevention of atherosclerosis by reducing the development of foam cells, repairing the impaired autophagy flux by significantly increasing the level of LC3-II, the number of auto phagolysosomes, the inhibition of PI3K-Akt-mTOR signaling, and the degradation of p62 in oxidized low-density lipoprotein-treated THP-1 cells^46^.

These studies were consistent with our observations that described the correlation between LC3-B and mTOR levels. The histopathological results of liver tissue by electron microscope were parallel to and supported the biochemical results. It is noticed that the CAME administration in different three-way (groups treated, prevented, and protected) manners exerted a beneficial effect on rat livers, where coffee ameliorated changes induced by Tet. However, our results suggested a better effect of CAME against NASH in preventive group. Moreover, as supportive evidence computerized analysis revealing detailed mapping of CGA’s interactions against key residues was carried out which provides a molecular basis for rational drug design. Specifically, the ability of the phenolic groups to interact with Asp 2357 (the metal-coordinating residue) suggests an impact on catalysis beyond simple ATP exclusion, potentially stabilizing an inactive or metal-displaced conformation of the enzyme. Future medicinal chemistry efforts could focus on simplifying the quinic acid portion of CGA while retaining or enhancing the phenolic functionality to improve synthetic tractability and optimize the binding energy^47^. This data-driven approach, leveraging the natural product scaffold, is key to developing novel, highly effective kinase inhibitors. The present findings demonstrated that CAME modulated autophagy-related markers, as evidenced by alterations in LC3B and mTOR expression. However, since autophagic flux was not directly assessed and phosphorylated mTOR levels were not measured, the results should be interpreted as an association with autophagy pathway modulation rather than definitive evidence of direct autophagy induction. Therefore, further mechanistic studies are required to clarify the dynamic regulation of autophagy in the observed hepatoprotective effect.

## Conclusion

Supplementation with *Arabica coffee* methanolic extract enhanced protective defense against NASH and was associated with the modulation of autophagy-related markers, including LC3B and mTOR expression. These findings might suggest a potential involvement of autophagy pathway regulation in the hepatoprotective effect of Arabica coffee. Even though the current study explored the involvement of autophagy modulation in the protective effect of Arabica coffee against NASH through evaluation of LC3B and mTOR expression, autophagic flux was not directly assessed, and phosphorylated mTOR was not evaluated, thus more investigations incorporating particularly on isolated bioactive constituents such as chlorogenic acid in addition to the incorporation of dynamic autophagy specific markers are recommended to further elucidate the mechanistic role of autophagy in the observed hepatoprotective effect along with better understanding their molecular interactions and therapeutic potential against NASH.

## Data Availability

Data is provided within the manuscript.
